# Comparison of clinico-microbiological profile and treatment outcome of in-house and referred post cataract surgery endophthalmitis in a tertiary care center in South India

**DOI:** 10.1186/s12348-016-0113-0

**Published:** 2016-11-24

**Authors:** Vikas Ambiya, Taraprasad Das, Savitri Sharma, Jay Chhablani, Vivek Dave, Subhadra Jalali, Raja Narayanan, Joveeta Joseph

**Affiliations:** 1 Srimati Kanuri Santhamma Retina Vitreous Center, Kallam Anji Reddy Campus, LV Prasad Eye Institute, Hyderabad, India; 2 Jhaveri Microbiology Center, Brien Holden Research Center, LV Prasad Eye Institute, Hyderabad, India; 3 LV Prasad Eye Institute, Road No. 2, LV Prasad Marg, Banjara Hills, Hyderabad, 500034 India

## Abstract

**Background:**

The purpose of the study is to compare the clinico-microbiological profile and treatment outcome of in-house vs referred cases of post cataract surgery endophthalmitis in a tertiary eye care facility in South India.

**Methods:**

The clinical records of 50 culture-positive cases each of in-house (group A) and referred (group B) post cataract surgery endophthalmitis were analyzed. The management protocol was similar in both groups.

**Results:**

The time to report to the institute was longer in group B (group B 13.63 [±11.67; 95% CI, 9.95–17.31] days; group A 6.83 [±7.61; 95% CI, 4.57–9.09] days; *P* = 0.002). The average inflammatory scores in presentation were comparable (group A 17.85 ± 5.83; group B 18.18 ± 7.35; *P* = 0.243). The final visual outcome was clinically superior in group A (≥20/200-group A 60.42% and group B 44%, *P* = 0.11; ≤20/400-group A 37.5% and group B 52%, *P* = 0.62), but statistically not significant. There were more gram-positive organisms in group A (62% vs 38%; *P* = 0.027) and more gram-negative organisms in group B (52% vs 24%; *P* = 0.007). Gram-positive bacteria were mostly sensitive to vancomycin (95.24% to 96.67%), but gram-negative bacteria were partly sensitive to ceftazidime (58.33% to 64%).

**Conclusions:**

One could suspect gram-negative infection more often in the referred cases of endophthalmitis. While vancomycin could continue to be the antibiotic of choice in gram-positive bacteria, specific antibiotic following due sensitivity for gram-negative bacteria should replace the empiric use of ceftazidime.

## Background

Patients with post cataract surgery endophthalmitis treated by us at a tertiary eye care facility in Hyderabad, India, are a pool of “in-house” and “referred” cases for further management. A decade and half ago, we reported the microbiological profile of these pooled patients [1] in which the gram-positive cocci accounted for less than 50% eyes and gram-negative bacilli were detected in more than 25% eyes. This was contrary to the report of the Endophthalmitis Vitrectomy Study (EVS) [2] results published before our report and also is contrary to the European Society of Cataract and Refractive Surgery (ESCRS) [3] study results published after our report. In view of these differences, we reexamined the microbiological profile and antibiotic sensitivity between two distinct groups of patients, in-house and referred, managed at this facility. We hypothesized that the in-house endophthalmitis profile (both clinical and microbiological) and outcome after treatment could be different.

## Methods

The clinical records of all culture-positive cases of post cataract surgery endophthalmitis managed at our institute between January 2010 and December 2014 were reviewed. There were 50 cases of in-house (group A) endophthalmitis. These 50 cases were matched with the first 50 consecutive culture-positive referred cases in the same period (group B; beginning December 2014 backwards and ending March 2010). Two cluster endophthalmitis cases, one in-house (reported earlier) [4] and one referred (reported by another treating center) [5] were excluded from both groups. Institutional review board approval (LEC 09-15-110) was obtained for retrospective data collection and analysis.

A detailed ocular history (cataract surgery, event to onset of symptoms, time to presentation, and previous treatment history in referred cases), the demography (age, gender), laterality, systemic factors (diabetes and immune status) were recorded. Management of all these patients was as per the institutional protocol. Briefly, this consisted of a comprehensive clinical examination (uncorrected and best corrected visual acuity, slit lamp biomicroscopy, applanation/digital tonometry, indirect ophthalmoscopy) and ultrasonography. The severity of inflammation in presentation was measured in all cases by our earlier published inflammatory score [6]. Briefly, this consisted of slit lamp and indirect ophthalmoscopic features of the cornea, anterior chamber, iris, and vitreous in a scale of 0 (no involvement) to 4 (gross involvement) and additional allowance given for any opaque ocular tissues not allowing further examination. The treatment consisted of intraocular antibiotics (vancomycin 1 mg in 0.1 ml normal saline; ceftazidime 2.25 mg in 0.1 ml normal saline) with either vitreous biopsy or core vitrectomy. This was followed with topical antibiotics (every 2 h), topical prednisolone acetate 1% (every 4 h) except in the eyes with significant corneal infiltrate and cycloplegic (every 8 h). The use of intravitreal corticosteroid (typically, dexamethasone 0.4 mg in 0.1 ml) and systemic antibiotics (typically, oral ciprofloxacin 1500 mg/day in two divided doses, in adult) were left to the discretion of the treating faculty. The vitreous sample was plated on the same day for culture. Microbiological work-up of undiluted vitreous included microscopy (after vital dye staining), culture (aerobic, anaerobic, and fungus), and antibiotic sensitivity (Kirby-Bauer disc diffusion). Unlike the current institutional standard, the polymerase chain reaction (PCR) was not done routinely in this period. Any further administration of intravitreal antibiotics with or without corticosteroid was guided by the culture report of the vitreous sample and the sensitivity of the cultured microorganism.

### Statistical analysis

The ETDRS visual acuity was converted to logMAR equivalent for statistical analysis. In each group, the mean values of best corrected visual acuity (BCVA) at baseline and at the last follow-up were compared by analysis of variance (ANOVA). The mean values detected in the two groups at each time point were compared by *t* test. The sensitivity profile of the microorganisms cultured in the two groups was analyzed using the Fisher’s exact test. Values of *P* < 0.05 were considered statistically significant. The two in-house endophthalmitis patients were excluded from the analysis for final visual outcome and inflammatory score because they received Descemet’s stripping automated endothelial keratoplasty along with cataract surgery but were included in the analysis for microbiological profile.

## Results

### Baseline characteristics

There were 50 cases each in either group. Between the two groups, there was no statistically significant difference in the demographic profile and in the diabetes status. There was a statistically significant difference between the two groups in the time to report to the institute with endophthalmitis (group A 6.83 [±7.61; 95% CI, 4.57–9.09] days; group B 13.63 [±11.67; 95% CI, 9.95–17.31] days; *P* = 0.002). There was no significant difference in average inflammatory score at presentation (group A 17.85 ± 5.83; group B 18.18 ± 7.35; *P* = 0.243). These details are shown in Table 1.

**Table 1:** Baseline characteristics and final visual outcome

	Group A. In-house (n = 50)	Group B. Referred (n = 50)	P value
Mean age (years) ± SD	58.13 ± 12.81	58.74 ± 13.29	0.41
Mean baseline BCVA logMAR (ETDRS equivalent) ± SD	1.99 (20/1954) ± 0.61	1.96 (20/1824) ± 0.45	0.48
Male: Female	29: 21	26: 24	0.55
Mean event to report time days (± SD, 95% CI)	6.83 (±7.61; 95% CI, 4.57 – 9.09)	13.63 (±11.67; 95% CI, 9.95 – 17.31)	0.002*
Number of diabetics	6	2	0.14
Inflammatory score (Mean ± SD)	17.85 ± 5.83	18.18 ± 7.35	0. 24
Final best corrected visual acuity			
≥ 20/200	29 of 48 (60.42%; 95% CI, 45.30-73.89)	22 of 50 (44%; 95% CI, 30.27-58.65)	0.11
≤ 20/400	18 of 48 (37.5%; 95% CI, 24.32-54.67)	26 of 50 (52%; 95% CI, 37.58-66.12)	0.62

*Statistically significant

### Visual outcome at last visit

The patients were followed up for a mean duration of 7.14 (±6.41; range 1.03–44.3) months in group A and 5.69 (± 5.27; range 1.0–28.73) months in group B (*P* = 0.37). The improvement in visual acuity of 0.719 logMAR in group A (approximately 7 ETDRS lines; 95% CI, 0.41–1.03, *P* < 0.001) and of 0.528 logMAR in group B (approximately 5 ETDRS lines; 95% CI, 0.32–0.74, *P* = 0.001) was statistically significant, but not significantly different between the two groups (*P* = 0.197). In general, the patients in group A (in-house) had better visual recovery than group B (referred) ≥20/200 in 60.42% (95% CI, 45.30–73.89) instances in group A vs 44% (95% CI, 30.27–58.65) instances in group B, and ≤20/400 in 37.5% (95% CI, 24.32-54.67) instances in group A and 52% (95% CI, 37.58–66.12) instances in group B. These were, however, only clinically significant (Table 1).

### Microbiological profile

The microbiological profile is shown in Table 2. Group A had more gram-positive bacteria (*n* = 31/50, 62%; 95% CI, 47.16–75.00 in group A vs *n* = 19/50, 38%; 95% CI, 25.00–52.84 in group B; *P* = 0.027) and there were more gram-negative bacteria in group B (*n* = 12/50, 24%; 95% CI, 13.52–38.49 in group A vs *n* = 26/50, 52%; 95% CI, 37.58–66.12 in group B; *P* = 0.007). Statistically, there was no significant difference in the number of fungal infections (*n* = 4/50, 8%; 95% CI, 2.59–20.11 each, in groups A and B; *P* = 1) or in the number of mixed infections (*n* = 3/50, 6%; 95% CI, 1.56–17.54 in group A vs *n* = 1/50, 2%; 95% CI, 0.1–12.01 in group B; *P* = 0.617).

**Table 2:** Microbiological spectrum

Isolate			Number	
			Group A In-house (N = 50)	Group B Referred (N = 50)
Gram positive (n = 31 in A, n = 19 in B, P = 0.027^a^)	*Staphylococcus*	Coagulase negative *Staphylococcus*	5	9
		*Staphylococcus epidermidis*	7	2
		*Staphylococcus aureus*	5	0
		Total	17	11
	*Streptococcus*	*Streptococcus pneumoniae*	9	2
		*Streptococcus* species	2	1
		Total	11	3
	Others	*Bacillus* species	3	1
		*Corynebacterium species*	0	2
		*Nocardia species*	0	2
		Total	3	5
Gram negative (n = 12 in A, n = 26 in B, P = 0.007^a^)	*Pseudomonas*	*Pseudomonas aeruginosa*	8	11
		*Pseudomonas* species	0	5
		Total	8	16
	Others	*Enterobacteriaceae*	4	4
		Other Gram negative bacteria	0	6
		Total	4	10
Others	Fungi	Fungi	4	4
	Mixed infections	Multiple bacteria	1	1
		Bacterium + Fungus	2	0
		Total	3	1

^a^Statistically significant

### Antibiotic-sensitivity profile

**Fig. 1:**
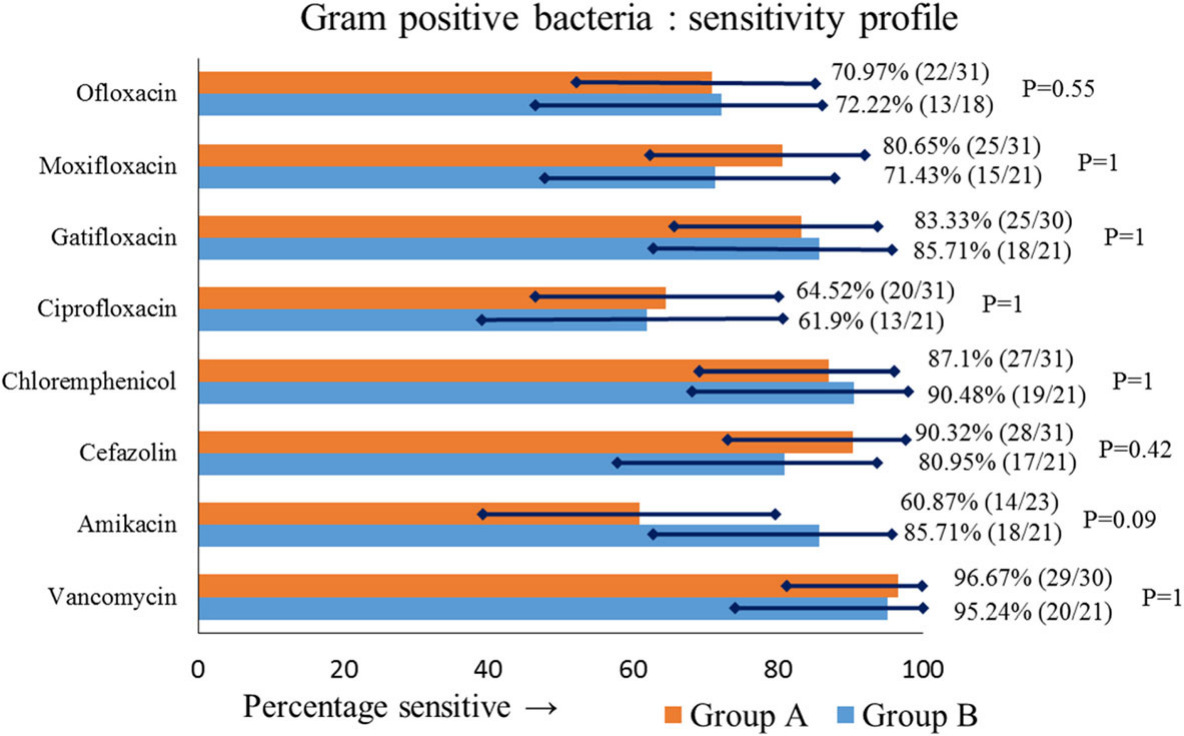
Antibiotic sensitivity profile of gram-positive isolates in groups A and B, showing percentage sensitivity with 95% CI

**Fig. 2:**
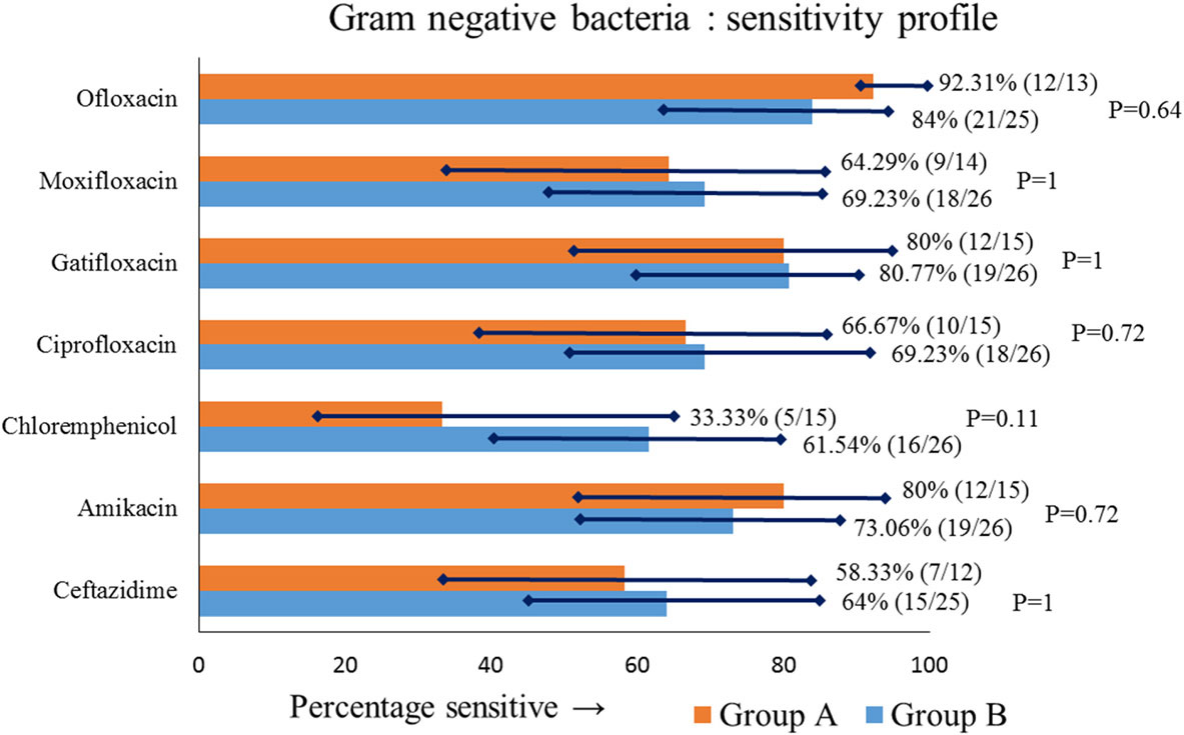
Antibiotic sensitivity profile of gram-negative isolates in groups A and B, showing percentage sensitivity with 95% CI

The sensitivity profile of the bacteria in the two groups is shown in Figs. 1 and 2. More than 95% gram-positive isolates in both groups (96.67% in group A, 95.24% in group B; *P* = 1) were sensitive to vancomycin and between 85% and 90% (90.32% gram-positive isolates in group A, and in 85.71% in group B) were sensitive to cefazolin. Only 58.33% of gram-negative isolates in group A and 64% gram-negative isolates in group B were sensitive to ceftazidime. The sensitivity of amikacin compared to ceftazidime in both groups (group A: amikacin 80% vs ceftazidime 58.33% sensitive, *P* = 0.22; group B: amikacin 73.06% vs ceftazidime 64% sensitive, *P* = 0.56) was not statistically significant.

### Ceftazidime resistance

In group A, 41.67% (5/12) gram-negative isolates were resistant to ceftazidime and, of these five resistant cases, only 40% (2/5) were sensitive to amikacin, but all were sensitive to imipenem. The 3 amikacin resistant isolates in group A belonged to *Pseudomonas* species. In group B, 36% (9/25) gram-negative isolates were resistant to ceftazidime, and of these, 77.78% (7/9) were sensitive to amikacin (Table 3).

**Table 3:** Antibiotic sensitivity profile of ceftazidime-resistant gram- negative isolates in groups A and B

S No.	Endophthalmitis isolate	Amikacin	Imipenem	Chloramphenicol	Ciprofloxacin	Gatifloxacin	Moxifloxacin	Ofloxacin	Tobramycin
Group A									
1	*Eschericia coli*	I	S	S	R	I	R	R	NT
2	*Enterobacter cloacae*	S	S	S	I	S	I	S	R
3	*Enterobacter cloacae*	S	S	R	I	S	I	S	R
4	*Pseudomonas aeruginosa*	R	S	R	R	R	R	NT	NT
5	*Pseudomonas aeruginosa*	R	S	R	R	R	R	NT	NT
Group B									
1	*Achromobacter xylosoxidans*	S	NT	S	S	S	S	S	S
2	*Eschericia coli*	S	NT	S	S	S	S	S	S
3	*Klebsiela pneumoniae*	S	S	R	R	R	R	R	S
4	*Pseudomonas aeruginosa*	R	S	R	R	R	R	R	R
5	*Pseudomonas species*	S	NT	S	S	S	S	S	S
6	*Pseudomonas stutzeri*	R	NT	S	S	S	R	S	R
7	*Pseudomonas stutzeri*	S	R	R	R	R	R	R	S
8	*Sphingomonas paucimobilis*	S	NT	S	R	S	R	S	S
9	*Stenotrphomonas maltophilia*	S	NT	S	S	S	S	S	S

*I* intermediate sensitivity, *NT* not tested, *S* sensitive, *R* resistant

### Prior therapy

Five patients had received prior therapy before being referred to us (Table 4). This included four adults and one child. All of them had received vitreous biopsy/core vitrectomy and repeated intravitreal antibiotics. The presenting vision was between light perception (*n* = 3) to ambulatory vision. Three of them grew *Pseudomonas aeroginosa*. For the following further treatment, none of the eyes infected with *P. aeruginosa* recovered vision. Two of the five patients, one infected with *Rhizobium radiobacter* and one infected with *Candida pelliculosa* recovered to 6/36 and 6/15, respectively.

**Table 4:** Baseline profile and outcome of patients with intervention prior to referral (group B)

S no.	Age (years)	Prior intervention	Presenting BCVA	Our intervention	Organism	Sensitivity	Final BCVA
1	70	IOAB	LP	PPV + 2x IOAB	*P. aeroginosa*	Sensitive to amikacin, ceftazidime, gatifloxacin, moxifloxacin, cirprofloxacin, and ofloxacin Resistant to chloramphenicol	LP
2	10	2x IOAB	LP	PPV + 2x IOAB	*Streptococcus pyogenes*	Sensitive to vanomycin, gatifloxacin, moxifloxacin, cirprofloxacin, and ofloxacin Resistant to amikacin	
3	64	AC wash + IOAB	LP	Vitreous biopsy + 5x IOAB	*P. aeruginosa*	Sensitive to amikacin, ceftazidime, gatifloxacin, moxifloxacin, cirprofloxacin, and ofloxacin Resistant to tobramycin	LP
4	62	Vitreous biosy + IOAB	6/36	PPV + 2x IOAB	*R. radiobacter*	Sensitive to amikacin, ceftazidime, gatifloxacin, moxifloxacin, cirprofloxacin, ofloxacin Resistant to chloramphenicol	6/36
5	55	2x IOAB	6/36	PPV + IOAB + 2x Intravitreal Amphoterecin B	*C. pelliculosa*	—	6/15

*IOAB* intraocular (antibacterial) antibiotic injection

## Discussion

Endophthalmitis, the most dreaded complication following cataract surgery, has an incidence from 0.04% to 0.13% [7–11]. Gram-positive bacteria were the predominant isolates in the EVS [2] and the ESCRS [3] studies. We and others in India have reported different microbiological profile of infection where the gram-positive cocci infection was relatively less, and gram-negative bacilli infection was relatively more common [1, 12]. These reports included both in-house and referred cases of endophthalmitis. Two Indian reports have published the in-house endophthalmitis data [13, 14]. In our report [14], culture-positive endophthalmitis was 0.07%; in this cohort 64.8% (*n* = 24/37) grew gram-positive cocci (*n* = 16/37, 43.2% were *Staphylococcus epidermidis*) and 24.3% (*n* = 9/37) grew gram-negative bacilli (*n* = 5/37, 13.5% were *P. aeruginosa*). In the other report, [13] clinical endophthalmitis was 0.09%, but the microbial profile was not part of this report.

Our hypothesis that the profiles of the two groups of endophthalmitis, in-house and referred could be different, was only partly true. There were differences in two areas—one, in the reporting time and two, in the microbiological spectrum. The delay in reporting time of the referred patients was likely because it depended on the referring ophthalmologist. The detection of more gram-positive cocci in the in-house cases and more gram-negative bacilli in referred cases was indeed a surprise (Table 2). The microbial spectrum of in-house endophthalmitis was closer to the EVS [2] and ECRS [3] reports. It is possible that only more fulminant cases that did not respond to initial treatment, invariably caused by gram-negative or similar virulent organisms, were referred to our facility.

The antibiotic sensitivity profile was nearly similar to our earlier report. Gram-positive bacilli had good sensitivity to vancomycin in either group, similar to the EVS report [2], and the gram-negative bacilli had poor sensitivity to ceftazidime in either group, similar to our earlier report [1]. All ceftazidime resistant gram-negative bacilli in group A (in-house cases) were sensitive to imipenem. Additionally, 77.78% of resistant isolates from referred cases were sensitive to amikacin. Imipenem appears to be good choice in gram-negative organisms resistant to ceftazidime and amikacin [15–17]. Amikacin could still be an alternative choice, though we have already reported emergence of gram-negative bacteria resistant to both ceftazidime and amikacin [18]. Hence it is prudent to always do a culture-sensitivity test for appropriate management.

The limitations of this study included small number of cases (50 in either group), small number of individual isolates tested for sensitivity, and non-inclusion of PCR as routine basis. The strength of the study is the use of uniform management protocol in both groups. The study clearly demonstrates that the post cataract surgery infection pattern in India may not be significantly different from the ones reported by the EVS and ESCRS reports, though one must employ wiser discretion in treating the in-house and referred patients in a referral facility such as ours.

## Conclusions

The study confirms that the gram-positive bacteria are the most common in post cataract surgery endophthalmitis in both in house and referred cases, but the gram-negative bacteria are more common in the referred cases in the teaching tertiary eye care facility in India. *P. aeruginosa* is the common gram-negative bacteria, and increasingly, they are resistant to commonly used antibiotic, ceftazidime. Hence, a microbiology and antibiotic sensitivity is mandatory while the EVS-based empiric treatment of post cataract surgery endophthalmitis treatment could continue for the time being. It is necessary to weigh the clinical response vis-a-vis the culture sensitivity to make a further decision. This implies seeing the patient 3–4 days after the initial treatment.
